# Explant pathology in Biliary Atresia post Kasai procedure: a tale of two livers

**DOI:** 10.4322/acr.2024.521

**Published:** 2024-10-08

**Authors:** Sunayana Misra, Sonia Badwal, Shashi Dhawan, Arpita Mittal, Naimish Mehta, Nishant Wadhwa, Arjun Maria

**Affiliations:** 1 Sir Ganga Ram Hospital, Department of Pathology, New Delhi, India; 2 Sir Ganga Ram Hospital, Department of Surgical Gastroenterology and Liver Transplantation, New Delhi, India; 3 Sir Ganga Ram Hospital, Pediatric Gastroenterology and Hepatology, New Delhi, India

**Keywords:** Neonatal cholestasis, Biliary atresia, Cholangitis, Portal hypertension, Hepato-porto-enterostomy

## Abstract

Biliary atresia (BA) is a progressive inflammatory cholangiopathy of infancy that results in fibrous obliteration of the extrahepatic and intrahepatic bile ducts. In untreated patients, this leads to biliary-type cirrhosis within the first two years of life. Timely diagnosis of BA with a lack of significant hepatic fibrosis is critical and surgical drainage (Kasai procedure) within the first two months of life is the initial treatment modality with the highest success rate. Ultimately, liver transplantation is required due to surgical drainage complications, such as recurrent cholangitis, failure to thrive, and portal hypertension (PHTN). Histopathological findings of hepatectomy specimens after failed and successful Kasai procedures are vastly different depending on the subsequent course of liver disease. Bile flow is inadequate following a failed Kasai procedure with rapid development of biliary cirrhosis. Explants from patients with successful Kasai procedure may show cholestatic (recurrent cholangitis), vascular (obliterative venopathy, regenerative hyperplasia, and PHTN), or an interplay of both cholestatic and vascular abnormalities. Pathologists need to be aware of explant histopathology (post-successful Kasai procedures) with a clinical course dominated by PHTN for precise documentation of vascular abnormalities.

## INTRODUCTION

Biliary atresia (BA) is a progressive inflammatory cholangiopathy of infancy resulting in fibrous obliteration of the extrahepatic and intrahepatic bile ducts.^1^ The incidence of BA ranges from 1 in 9000 to 1 in 15000 live births.^2^ The affected children, if untreated, develop biliary-type cirrhosis within the first two years of life.

Timely diagnosis of BA is critical due to rapid progression toward cirrhosis. Surgical drainage {Kasai procedure or hepatic-porto-enterostomy (KPE)}, the only effective intervention, is successful in approximately half of the patients.^3^ Early diagnosis of BA, lack of significant fibrosis, and KPE performed before two months of life have the highest success rates.^4^ If the initial surgery does not restore bile flow, liver transplantation (LT) is lifesaving, making BA the most frequent indication for LT in children.^5^

Histopathological findings of hepatectomy specimens after failed and successful KPE are vastly different depending on the subsequent course of liver disease. Bile flow is inadequate following a failed KPE with rapid development of biliary cirrhosis. Portal hypertension (PHTN) and recurrent cholangitis are the most common indications for LT later in life.^6^ We present two BA patients who underwent KPE at the same age; however, the outcomes were very different. We emphasize gross and histopathological findings in the explant liver after unsuccessful and successful Kasai procedures.

## CASE REPORTS

### Patient 1

A 6-year-old female patient who was diagnosed with BA at 2 months of age underwent LT due to growth failure and PHTN. She had presented to the hospital with neonatal cholestasis and conjugated hyperbilirubinemia on day 60 of life. Diagnostic biopsy was consistent with an obstructive biliary disease [BA with F2 fibrosis stage; Batts-Ludwig staging]. An intraoperative cholangiogram performed favored BA, and she underwent KPE on day 63 of life. For the ensuing 5 years, she was well, anicteric with near-normal serum bilirubin levels. No prior episodes of cholangitis were reported.

Currently, she has presented with two episodes of hematemesis one year and six months prior, with grade II esophageal varices for which endoscopic band ligation was performed. On physical examination, stigmata of chronic liver disease (CLD) in the form of spider nevi and palmar erythema were noted. The abdomen was distended and nontender with visible dilated veins and an everted umbilicus. There was mild splenomegaly. Ultrasound of the abdomen revealed coarse nodular echotexture of the liver with PHTN. Triphasic CT angiography was within normal limits. Her biochemical (liver and renal function tests) and hematological parameters were within normal limits. A sepsis workup and viral serology were negative. An ABO-compatible living donor LT (LDLT) was performed, and her mother served as the donor.

**Figure 1:**
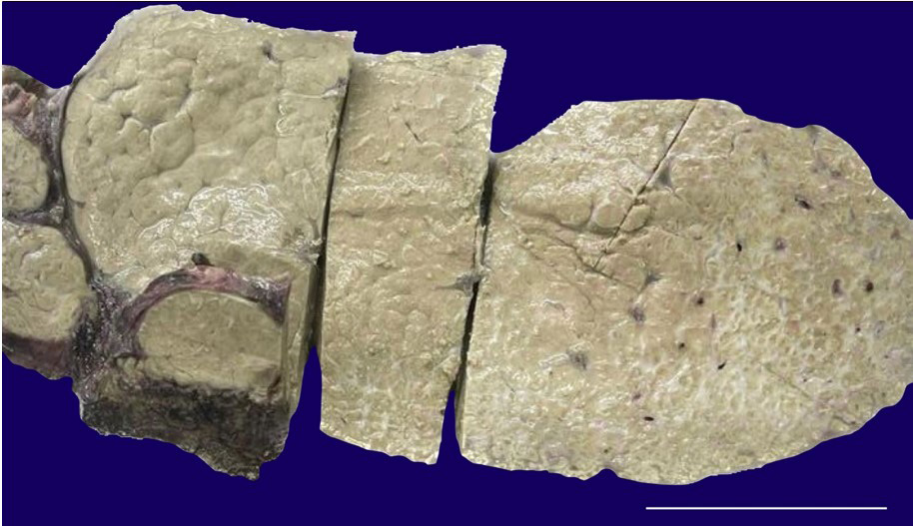
Gross view of the patient’s 1 explant liver showing a tan color of the hepatic parenchyma with large, vague, perihilar nodules and increased gray‒white fibrous streaks at the periphery (scale bar= 5 cm).

Grossly, the hepatectomy specimen weighed 498 grams. The outer surface was smooth to irregular, with sharp margins. The cut surface of the liver was pale and tan in color, with atrophy of the peripheral areas and hypertrophy of the central areas. Very large to large, irregular, incomplete, partial nodules were observed toward the hilum, while the periphery showed mixed nodularity (Figure 1).

**Figure 2:**
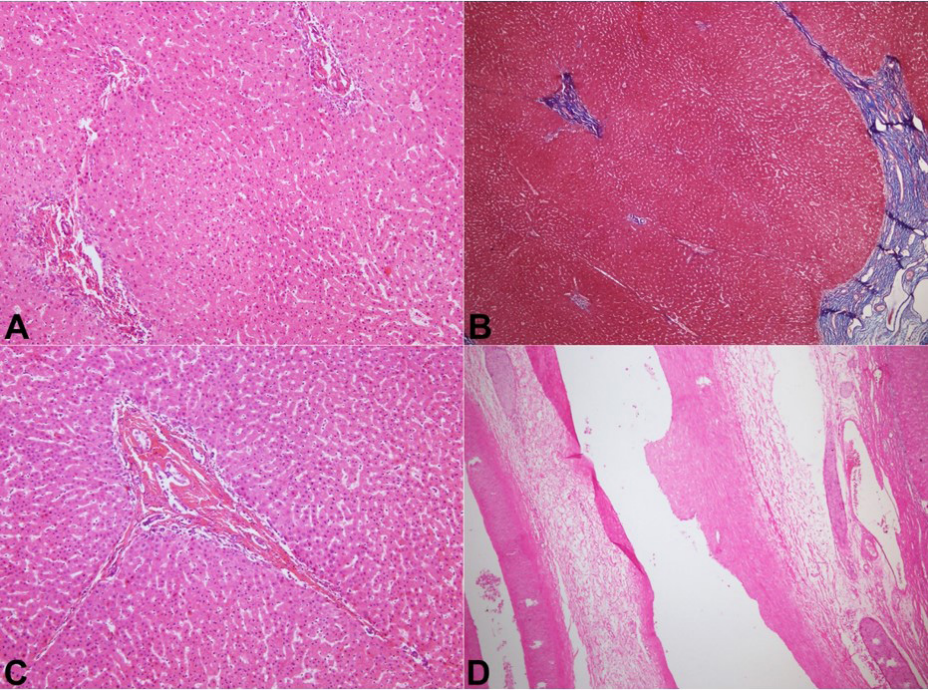
Photomicrographs of the liver – explant Patient 1 – **A** and **B –** The perihilar (central) liver parenchyma shows nearly normal maintained noncirrhotic liver architecture (**A** -HE, 40x, **B –** Masson trichome 40X); **C –** Portal tract showing dense stromal collagen, an intact native bile duct and an obliterated portal vein (HE, 200x); **D –** Dilated portal vein at the hilum with mild fibro-intimal hyperplasia (HE, 40x).

On microscopy, the hepatic parenchyma showed distortion of the lobular architecture due to the presence of macro regenerative nodules with advanced fibrosis toward the periphery. In contrast the central zones showed a predominantly maintained lobular architecture with partial nodular configuration and a few slender incomplete fibrous septae (Figures 2A, 2B). Sections from the peripheral zones showed incomplete to complete parenchymal nodules separated by fibrous septae with loose, perinodular, edematous fibrosis outlined by dense slings of collagen. The fibrous septae revealed minimal chronic inflammation. Few septae showed obliterated duct profiles with central scarred, fibrous stroma. Prominent arterial profiles and dilated to opened portal venous radicals were noted. Portal tracts within the larger macronodules showed native duct profiles with periductal sclerosis and mild peripheral ductular reaction. Central zones showed predominantly maintained lobular architecture with expanded portal tracts and irregular, intervening slender, perforated wisps of fibrosis (Figures 2A, 2B). A vague nodularity (without intervening fibrous bands) due to alternating hepatocyte cord atrophy and hypertrophy was observed, indicative of nodular regenerative hyperplasia (NRH). The portal tracts in these central areas showed dense portal stromal sclerosis, small bile ducts, prominent arterial profiles, and obliterated portal veins (Figure 2C). No prominent peripheral ductular proliferation was detected. There was no evidence of cholangitis. The central parenchyma also revealed scattered isolated arteries indicative of vascular flow abnormalities. No intraparenchymal/ductal cholestasis was observed. Sections from the hilum showed small, attenuated duct profiles with a periductal sclerotic collarette, mild medial hypertrophy of the hepatic artery, and mild fibromyxoid intimal hyperplasia of the dilated portal vein (Figure 2D). The final impression on the explant specimen was secondary biliary cirrhosis with nodular regenerative hyperplasia (NRH) of the central parenchyma and features of obliterative portal venopathy (OPV).

### Patient 2

**Figure 3:**
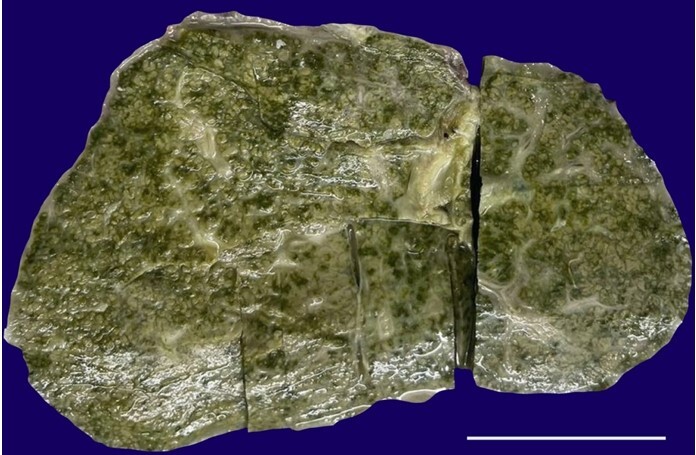
Gross view of patient’s 2 explant liver showing a bile-stained surface with biliary nodules (scale bar= 4cm).

An eight-month-old male child was taken up for LT due to failed KPE. He presented with neonatal cholestasis and was diagnosed with BA at 2 months of life, for which he underwent KPE. However, two months after the Kasai procedure, bilirubin levels did not normalize, and the patient remained deeply icteric. On examination, the abdomen was distended, and there was hepato-splenomegaly. Ultrasound revealed features of chronic liver disease. The sepsis work-up was negative. An ABO-compatible living donor LT (LDLT) was performed with the father serving as the donor. Grossly, the explant weighed 403 gm. The entire cut surface of the liver showed greenish bile-stained nodules ranging from 0.2 to 0.4 cm (Figure 3).

**Figure 4:**
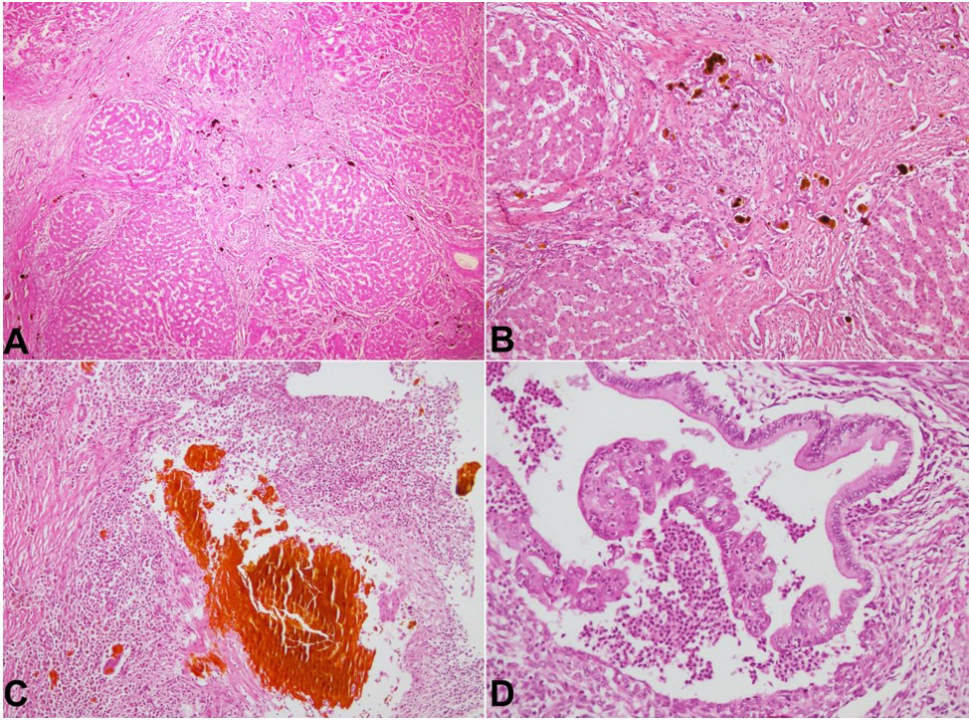
Photomicrographs of patient’s 2 explant liver. **A –** Distorted lobular architecture with fibrous bands dividing the liver parenchyma into irregular nodules (HE, 40x); **B** and **C –** The fibrous septae show a prominent peripheral ductular reaction, with many showing inspissated bile plugs (HE 100x); **C –** Hilum showing ulcerated bile duct profiles with intraluminal neutrophilic abscesses and bile lakes with surrounding bile pigment-laden xanthogranulomatous response (HE200x); **D –** Large bile duct at the hepatic hilum with neutrophilic duct damage (HE, 100x).

The hilum showed dilated bile ducts filled with inspissated bile. Histopathology revealed lobular distortion due to irregular jig-saw-shaped predominant micro- to few macro regenerative nodules with intervening, variable, irregular fibrous septae (Figure 4A). The fibrous septae showed mixed inflammation and prominent peripheral ductular reactions with bile plugs (Figure 4B). Hepatocytes showed prominent hepatocanalicular cholestasis, focal giant cell transformation, and cholestatic pseudorosettes. Sections from the hilum showed a large bile duct profile with an ulcerated lining and intraluminal neutrophilic abscesses (Figure 4C). Bile lakes and biliary excrescences were observed with a surrounding bile pigment-laden xanthogranulomatous response (Figure 4D). The final impression on the explant was that of secondary biliary cirrhosis with cholangitis and bile infarcts.

## DISCUSSION

BA is a fibro-sclerosing cholangiopathy of infancy that affects both the extrahepatic and intrahepatic bile ducts to a variable extent, eventually destroying the biliary tract.^1^ Without surgical intervention, disease progression leads to hepatic fibrosis, cirrhosis with liver failure, and death within 2 to 3 years of life.^1,4^ It classically presents during the neonatal period with cholestatic jaundice, acholic stools, and hepatomegaly in an apparently healthy infant.

Native liver survival (NLS) in BA patients depends on several factors, the most important of which is age at diagnosis, which determines the extent of liver fibrosis.^7^ Timely KPE, performed before 2 months of age, is aimed at restoring bile flow and attempts to reverse (to a certain extent) as well as prevent further cholestatic damage in these patients. In successful KPE, conjugated bilirubin is normalized within 4–6 months of the procedure^7^. Episodes of recurrent cholangitis determine a further course of the liver; the onset, severity and complications of PHTN; and faltering growth.

The Japanese BA registry reported that the prevalence of 20-year NLS post-KPE was 49%^8^. Parolini et al^9^ reported an NLS of 17.8% in BA patients over 20-year.^9^ In their study, Patel et al^6^ reported an NLS of 20.6% after KPE over 9 years.^6^ Moreover, pathologists are less commonly encountering post-Kasai-LT specimens, especially in India. This paper aims to document and analyze the gross and microscopic findings of two BA patients with very different outcomes, post-successful and post-unsuccessful KPE.

Several groups have performed histological studies of bile duct remnants removed during KPE in an attempt to predict NLS, among which the Gautier and Eliot systems have gained some popularity.^10^ The authors evaluated biliary remnants at three levels: the porta hepatis, the junction of the cystic and common hepatic ducts and an intermediate level. However, these classification systems are of historical importance only and do not correlate with NLS. In individual cases, serial sectioning often shows atretic ducts alternating with variably destroyed ducts due to patchy disease involvement.

Explant pathology in failed KPE is similar to that in untreated BA and shows a dark-green bile-stained surface and irregular to fine nodularity (depending on the extent of cirrhosis). The absence of proper biliary drainage due to progressive intrahepatic ductopenia leads to diffuse biliary cirrhosis.^7^

In patients with successful KPE that reaches childhood to early adulthood, the majority develop portal hypertension as a long-term complication.^6^ Patients who are complicated by repeated episodes of cholangitis exhibit parenchymal cholestasis and biliary cirrhosis, whereas patients who present with PHTN exhibit perihilar regenerative hyperplasia. In their study, Patel et al^6^ observed that native livers of patients with successful KPE requiring LT for PHTN did not show diffuse cholestasis or micronodular biliary cirrhosis, which is typical of failed KPE. Instead, they showed OPV features characterized by parenchymal atrophy/regeneration, portal stromal and vascular changes, and predominantly intrahepatic and sometimes extrahepatic portal vein occlusions.^11^ The presence of large regenerative macronodules in the hilar region and central zones of noncirrhotic liver parenchyma with peripheral zones of fibrosis has also been reported in a few previous studies.^12^ This suggests that in patients with successful KPE and well-established biliary drainage, obstructive cholangiopathy is not the main driving force for advanced liver disease. Instead, findings have been attributed to vascular abnormalities due to differential perfusion of different zones of the liver. Few surgical studies have also documented abnormalities such as hypoplasia and fibrosis/thickening in extrahepatic portal veins in children with BA.^13^ Patel et al^7^ also noted intimal thickening of the extrahepatic portal vein in BA patients.^7^ The degree of portal vein abnormality may influence the onset, severity, and progression of PHTN in BA patients. In patients with perihilar hyperplasia, the central region is functional and has nearly normal histology, with bile ducts accompanying most of the hepatic artery branches in the portal tracts.

In patients with successful KPE, a longer duration of recurrent cholangitis affects advanced fibrosis more adversely than the duration or severity of PHTN. Recurrent cholangitis causes portal inflammation, bile plugging, biliary epithelial injury, and portal fibrous expansion leading to biliary cirrhosis.^7^ Conversely, a post-KPE course dominated only by PHTN will show delicate septal fibrosis in the large perihilar zones in the explant, similar to inflow-tract-vascular abnormalities. This finding was further substantiated by an MRI study of 32 patients with BA after KPE in which patients with >40% of the area of near-normal liver architecture at the hilum did not require LT.^14^ Long-term survival after KPE may depend on the anatomical extent of regenerative perihilar hyperplasia and the ability of the perihilar region to maintain liver function in the presence of progression to cirrhosis in peripheral parts of the liver. In our study, patient 1 was complicated by PHTN and exhibited a perihilar regenerative noncirrhotic region (centered on segment 4) and peripheral biliary cirrhosis. The central region had nearly normal histology with features of OPV in the portal tracts.

Portal venous changes may play a role in the pathogenesis of BA. Some BA patients with extrahepatic portal vein obstruction may show portal cholangiopathy.^6^ Both intra- and extrahepatic bile ducts develop from mesenchymal signaling originating from the portal vein and its branches.^15^ Thus, intrinsic abnormalities in signaling of the portal vein mesenchyme may lead to both portal vein anomalies and bile duct developmental abnormalities.^15^

The explants of patients with successful KPE can show cholestatic (complicated by cholangitis), vascular (complicated by PHTN) or an interplay of both cholestatic and vascular abnormalities. For precise documentation of vascular abnormalities, pathologists must be aware of the findings in patients with successful KPE with a clinical course dominated by PHTN for precise documentation of vascular abnormalities.
